# Supplementary material for: Rapid turnover of life-cycle-related genes in the brown algae
Source: Genome Biol. 2019 Feb 14;20:35. doi: 10.1186/s13059-019-1630-6 (PMC6374913; doi:10.1186/s13059-019-1630-6)
Supplement: Supplementary file 3 — Protein alignments, corresponding Gblocks html file and CDS sequences for the lineage conserved gametophyte-biased genes. (ZIP 2109 kb) [file 13059_2019_1630_MOESM3_ESM.zip › Con_GA_alignments/Ec-12_006180.aa_ali.fasta-gb.htm]

Ec-12\_006180.aa.fasta.trans.aa\_ali.fasta


## Gblocks 0.91b Results

Processed file: **Ec-12\_006180.aa.fasta.trans.aa\_ali.fasta**  
Number of sequences: **2**  
Alignment assumed to be: **Protein**  
New number of positions: **377** (selected positions are underlined in blue)

```
                         10        20        30        40        50        60
                 =========+=========+=========+=========+=========+=========+
Ec-12_006180     ----------------------------------------------------------ME
MSTRG.35977.1    MAERGGHGRDGPVLPRDTIATHTGGAGAGGGNASRGVGAGLERERTSSGYKVGEEELPME
                                                                           ##


                         70        80        90       100       110       120
                 =========+=========+=========+=========+=========+=========+
Ec-12_006180     VPGRYEKMRVLGRGSFGAVTLVKDHRDSKLYAMKTLNCADKLEERVTAMAEVRLLKWLKH
MSTRG.35977.1    VPGRYEKMRVLGRGSFGAVTLVKDNRDSKLYAMKTLNCADKLEERVTAMAEVRLLKWLKH
                 ############################################################


                        130       140       150       160       170       180
                 =========+=========+=========+=========+=========+=========+
Ec-12_006180     PCVLTLFDVFLSADSRLVCLTTTYCESGDLAKIVKHASKTKSPLGEKTVLGWFAQLCLGV
MSTRG.35977.1    PCVVTLFDVFLSADGRLVCLTTTYCESGDLAKIVKHASKTKSLLGEKTVLGWFAQLCLGV
                 ############################################################


                        190       200       210       220       230       240
                 =========+=========+=========+=========+=========+=========+
Ec-12_006180     HNLHEEKKVLHRDIKPNNVFLMDSKKIVQLGDFGLAKVINDGDKQVKAEVGTPYYTSPEM
MSTRG.35977.1    HNLHEEKKVLHRDIKPNNVFLMDSKKIIQLGDFGLAKVLSDGDKQVKAEVGTPYYTSPEM
                 ############################################################


                        250       260       270       280       290       300
                 =========+=========+=========+=========+=========+=========+
Ec-12_006180     CNNQPYGFPSDVWSLGIVLYELLSLDVPFRSRDVVALVS---------------------
MSTRG.35977.1    CNNQPYGFPSDVWSLGIVLFELLSLDVPFRSRDVVALVSQVIKAPPPPLPEVYSDEVTAL
                 #######################################                     


                        310       320       330       340       350       360
                 =========+=========+=========+=========+=========+=========+
Ec-12_006180     ----------------QALACQPLRRHMSSFVKHYRPMNMAERQRRHDIKDLEAQVASII
MSTRG.35977.1    ARWMLQKDPLRRPTLSQALACQPLRRHMSSFVKHYRPMNMAERQRRHDIKDLEAQVASII
                                 ############################################


                        370       380       390       400       410       420
                 =========+=========+=========+=========+=========+=========+
Ec-12_006180     EHGGDDPQIPRDVTDGCGTLPAGAQDETATDTLNRADSKSHPGEARGGSVEGVEGAAAVL
MSTRG.35977.1    ERGGDDPQIPGDVNDGCGTCAQ--DDPAATDSPHRAGSRGQLLEAQGVGNEEAEGDAA--
                 ###################      #################################  


                        430       440       450       460       470       480
                 =========+=========+=========+=========+=========+=========+
Ec-12_006180     METAAGGGEGGFAAAAAAGRTRTADRSSVRSPSAISITADGIPVAARADEAENAGAAAAA
MSTRG.35977.1    LSAAAVGAEG--SETAPERGAVAVDRSAARSPSTAGPMLDGHVTALRTDEIEAS--TAAA
                                #####################################     ###


                        490       500       510       520       530       540
                 =========+=========+=========+=========+=========+=========+
Ec-12_006180     AATGGGGCPRCGARVPCACGGARGGGGGGGGNATPGRRFGSPQQSKGLRNVMSWATGGSI
MSTRG.35977.1    AAGCGGGCARCGARVPCACGSGV-------------------------------------
                 ####################                                        


                        550       560       570       580       590       600
                 =========+=========+=========+=========+=========+=========+
Ec-12_006180     TPPAGRGSEGDDGDSQDGSRSSNDVNRDGIERRRRSTDYEKGTTEWHSASSGGSVRSFSS
MSTRG.35977.1    ------------------------------------------------------------
                                                                             


                        610       620       630       640       650       660
                 =========+=========+=========+=========+=========+=========+
Ec-12_006180     VESFSKPSSSHGSRWRDRRKTGDGGKCSSSSPRDSGASIVGNGGDRKRSSRGTGFRKSEE
MSTRG.35977.1    ------------------------------------------------------------
                                                                             


                        670       680       690       700       710       720
                 =========+=========+=========+=========+=========+=========+
Ec-12_006180     GDDGADGGGDHETDSVDRSDNCLAAPARPKSPRSPRAHRSTRLKRVSGSSSAEASGCPDG
MSTRG.35977.1    ------------------------------------------------------------
                                                                             


                        730       740       750       760       770       780
                 =========+=========+=========+=========+=========+=========+
Ec-12_006180     GESEGEGMTAAVAAATTEESDSMRRRGSSNTGVTDKGYEDMGEGTPRPARSQPPGVDEST
MSTRG.35977.1    ------------------------------------------------------------
                                                                             


                        790       800       810       820       830       840
                 =========+=========+=========+=========+=========+=========+
Ec-12_006180     AVSAPADTTESNVALHEASPTEAGTSPTRRRQGDGAETEVSSKETGPGSPHLVAVEGGAS
MSTRG.35977.1    ------------------------------------------------------------
                                                                             


                        850       860       870       880       890       900
                 =========+=========+=========+=========+=========+=========+
Ec-12_006180     EKGIRQSGSSTSPAIRRSSTAAAAIALDRLVLTPRTPRSPRIVGSDSEPFKSAKRLSEAL
MSTRG.35977.1    ------------------------------------------------------------
                                                                             


                        910       920       930       940       950       960
                 =========+=========+=========+=========+=========+=========+
Ec-12_006180     TFAASSSEEDFLTGTATAAAGSTGPTPRRESGALGGALAIVEEAKGVREGMTVGGRVAEG
MSTRG.35977.1    ------------------------------------------------------------
                                                                             


                        970
                 =========+==
Ec-12_006180     RGNPAAVLGAPS
MSTRG.35977.1    -----------X
```

```
Parameters used
Minimum Number Of Sequences For A Conserved Position: 2
Minimum Number Of Sequences For A Flanking Position: 2
Maximum Number Of Contiguous Nonconserved Positions: 8
Minimum Length Of A Block: 10
Allowed Gap Positions: None
Use Similarity Matrices: Yes
```

```
Flank positions of the 5 selected block(s)
Flanks: [59  279]  [317  379]  [386  418]  [436  472]  [478  500]  

New number of positions in Ec-12_006180.aa.fasta.trans.aa_ali.fasta-gb:  377  (38% of the original 972 positions)
```
